# Mortality prediction in the elderly patients with coronary artery disease and atrial fibrillation: a retrospective machine learning approach

**DOI:** 10.3389/fmed.2026.1860173

**Published:** 2026-09-10

**Authors:** Yuyan Wang, Yangxun Wu, Yuting Zou, Shuai Mao, Yanzhu Yao, Yifan Wang, Yunzhang Zhao, Chao Lv, Li Fan, Tong Yin

**Affiliations:** 1 Institute of Geriatrics, Beijing Key Laboratory of Geriatric Multimorbidity Research, National Clinical Research Center for Geriatric Diseases, the Second Medical Center, Chinese PLA General Hospital, Beijing, China; 2 Medical School of Chinese PLA and Chinese PLA General Hospital, Beijing, China

**Keywords:** atrial fibrillation, coronary artery disease, elderly, machine learning, mortality

## Abstract

**Background:**

Elderly patients with coexisting coronary artery disease (CAD) and atrial fibrillation (AF) are at significantly increased risk of mortality. Accurate risk stratification is crucial for improving clinical management, yet a dedicated predictive tool for this specific population is lacking. The widely used CHA_2_DS_2_-VASc score demonstrates limited performance in predicting all-cause mortality in this complex comorbid group.

**Methods:**

A cohort of elderly inpatients (≥65 years) diagnosed with CAD and AF were retrospectively enrolled from the Department of Cardiology at the Chinese PLA General Hospital between January 2010 and December 2017. Baseline clinical data during hospitalization were collected, and all patients were followed up for all-cause mortality. Patients were randomly divided into a training set (70%) and a validation set (30%). Variable selection was performed using LASSO-Cox regression. Predictive models were established through a Cox proportional hazards model (via LASSO-Cox) to predict all-cause mortality risk. To assess model robustness and address potential overfitting, we performed 10-fold cross-validation and bootstrap resampling (500 replicates) with optimism-corrected C-statistics. Model performance was evaluated using the area under the receiver operating characteristic curve (AUC), calibration curves, and decision curve analysis (DCA), and compared against the CHA_2_DS_2_-VASc score.

**Results:**

A total of 1678 elderly patients with CAD and AF were randomly divided into a training set (*n* = 1174) and a validation set (*n* = 504). Through LASSO-Cox regression, 17 variables were identified as predictors associated with all-cause mortality. Two distinct models were developed: LASSO-Cox Model A included factors such as age, left ventricular ejection fraction, blood glucose, plasma fibrinogen, D-dimer, prothrombin time, NT-proBNP, hemoglobin, and hematocrit; LASSO-Cox Model B incorporated all variables from Model A plus acute myocardial infarction, history of myocardial infarction, renal insufficiency, heart failure, diabetes, stroke, chronic obstructive pulmonary disease, and malignant tumor.

The analysis of Cox proportional hazards regression models showed that in the training sets, the Area Under Curves (AUC) of model A, model B and CHA_2_DS_2_-VASc scores for predicting 1-year all-cause death were 0.83, 0.85 and 0.66, respectively; the AUC for 5-year all-cause death were 0.74, 0.76 and 0.62, respectively. In the validation sets, the AUC of model A, model B and the CHA_2_DS_2_-VASc score for predicting 1-year all-cause mortality were 0.79, 0.78 and 0.57, respectively; the AUC for 5-year all-cause death were 0.74, 0.75 and 0.57, respectively. The 10-fold cross-validation yielded a mean AUC of 0.83 (SD: 0.04) for 1-year mortality, and bootstrap resampling produced an optimism-corrected C-statistic of 0.679 (95% CI: 0.651–0.708), confirming that the model maintains clinically meaningful discriminative ability after accounting for optimism. Model B demonstrated better calibration and provided greater net clinical benefit in DCA.

**Conclusion:**

The LASSO-Cox machine learning model demonstrates superior predictive performance for all cause mortality relative to the traditional CHA_2_DS_2_-VASc score in elderly patients with CAD and AF.

## Introduction

Coronary artery disease (CAD) and atrial fibrillation (AF) are highly prevalent cardiovascular diseases in China, characterized by an increasing incidence with advancing age (1, 2). CAD is a leading cause of mortality in both developing and developed countries. As an age-related arrhythmia, AF is associated with a significantly elevated risk of death, with approximately half of all deaths in AF patients being attributable to cardiac causes (3). CAD and AF share common risk factors and pathophysiological features and therefore often coexist. The population aged 65 and above in China has risen to 264 million, accounting for 18.70% of the total population. The prevalence of comorbidities in the elderly, including these conditions, is increasing along with the accelerating aging of the population. Patients with concomitant CAD and AF have a significantly higher rate of adverse cardiovascular events compared to those with either condition alone (4, 5). Furthermore, among elderly patients (≥70 years), both the co-prevalence of these diseases and the risk of adverse clinical outcomes are markedly higher than in younger patients (6). The combination of CAD and AF in the elderly is associated with higher mortality (7), posing a severe threat to life and health. Consequently, predicting the all-cause mortality risk in elderly patients with CAD and AF is of paramount importance for clinical management.

The CHA_2_DS_2_-VASc score model is the most widely used clinical prediction tool for assessing thromboembolic risk and guiding antithrombotic therapy in AF patients (8). This scoring system can also predict the risk of adverse outcomes such as all-cause mortality and major adverse cardiac and cerebrovascular events (9–13). However, it has certain limitations in predicting adverse clinical outcomes specifically in elderly patients with coexisting CAD and AF (7). The 2022 European Society of Cardiology guidelines explicitly recommend that a systematic assessment of thrombotic risk in elderly patients with CAD or AF should integrate various types of risk predictors (14). Due to the widespread adoption of machine learning algorithms and their significant advantages in predictive performance, several studies have applied these algorithms to develop clinical models for predicting all-cause death in patients with either CAD or AF, demonstrating superior predictive value compared to traditional clinical prediction models. For instance, Chen et al. utilized LASSO regression and the random survival forest algorithm to select variables from 1,223 AF patients aged 55 and above. They employed six machine learning algorithms, including Cox regression, to build models predicting 1-year all-cause mortality. Their results indicated that the model using LASSO regression for variable selection performed the best, exhibiting higher predictive power and clinical net benefit than the traditional CHA_2_DS_2_-VASc model (15). Consequently, using LASSO regression to select clinical risk factors combined with machine learning algorithms to build all-cause mortality risk models has been proven effective and accurate in cardiovascular disease patients aged 50 and above, characterized by CAD or AF, and is significantly superior to traditional clinical prediction models. Among these approaches, combining variable selection via LASSO regression with a Cox proportional hazards model has been shown to achieve optimal results. LASSO regression is favored for variable selection due to its high accuracy and stability, and is commonly used in modeling for cancer, heart failure, and AF populations (16–19). Furthermore, the LASSO-Cox regression method builds upon LASSO regression by estimating the relationship between the selected variables and the time to endpoint events. It serves as a method to prevent model overfitting and has been widely incorporated into various types of machine learning algorithms (20–22).

Currently, there is a lack of established risk prediction models specifically for all-cause mortality in the elderly population with concomitant CAD and AF. Therefore, this study aims to address this gap by establishing a clinical follow-up cohort database and leveraging advanced big data analytics and machine learning model algorithms. It seeks to thoroughly investigate the performance of a risk assessment tool for all-cause mortality in this specific patient group. The development of such a model holds significant clinical utility and practical value for improving the prognosis of elderly patients with CAD and AF.

## Materials and methods

### Study population

A consecutive cohort of elderly patients diagnosed with both CAD and AF during hospitalization in the Department of Cardiology, Chinese People's Liberation Army (PLA) General Hospital, between January 2010 and December 2017, was enrolled. The patients were randomly divided into a training set and a validation set at a 7:3 ratio. During the dataset division, stratified sampling was employed to ensure the proportion of samples with and without the endpoint event was identical between the training and validation sets. Inclusion criteria: (1) Age ≥ 65 years; (2) Clinical diagnosis of CAD combined with AF. Exclusion criteria: (1) Inability to undergo follow-up for at least one year; (2) Missing crucial information in electronic medical records (including data on blood biochemistry and past medical history); (3) Participation in other clinical trials; (4) Terminal patients during the hospitalization period. This study was reviewed and approved by the Ethics Committee of the Chinese PLA General Hospital. Informed consent was obtained from all enrolled patients.

### Definition of disease

CAD was defined as stable coronary artery disease (including stable angina pectoris, prior myocardial infarction, and ischemic cardiomyopathy) (23) or acute coronary syndrome (including unstable angina and acute myocardial infarction) (24). AF was defined as a cardiac rhythm characterized by irregular, discrete P waves and irregular RR intervals (in the absence of atrioventricular block) recorded by a standard 12-lead electrocardiogram or a single-lead rhythm strip lasting >30 seconds (8), encompassing paroxysmal, persistent, or permanent AF.

### Collection of clinical information

Clinical data of the enrolled subjects were obtained from inpatient medical records or outpatient systems. The collected information included: (1) Demographics: Sex, age, etc. (2) Lifestyle factors: History of smoking and alcohol consumption, etc. (3) Past medical history and comorbidities: History of renal dysfunction, stroke, chronic obstructive pulmonary disease (COPD), peripheral arterial disease, malignant tumors, etc. (4) Previous medication history: History of cardiovascular medication use, etc. (5) CAD type: Stable angina, acute coronary syndrome (ACS) (including unstable angina, acute ST-segment elevation myocardial infarction, acute non-ST-segment elevation myocardial infarction), etc. (6) AF type: Paroxysmal AF, persistent AF, permanent AF. (7) Concomitant medications: Antiplatelet agents, anticoagulants, beta-blockers, calcium channel blockers, statins, amiodarone, etc. (8) Laboratory tests and examinations: Complete blood count, liver and kidney function tests, coagulation profile, left ventricular ejection fraction (LVEF), etc. (9) Interventional procedures and surgeries: Percutaneous coronary intervention (PCI), radiofrequency ablation, coronary artery bypass grafting (CABG), etc. (10) Discharge information: Discharge diagnoses and discharge medications. (11) In-hospital adverse events.

### Patient follow-up and endpoint events

All enrolled patients were followed for a minimum of one year. The primary endpoint of this study was all-cause mortality. All-cause mortality was defined and categorized according to the International Classification of Diseases, Tenth Revision (ICD-10) (25, 26), including both cardiac and non-cardiac death. Cardiac death was defined as death due to fatal myocardial infarction, sudden cardiac death, or stroke. Non-cardiac death referred to death attributable to causes other than those listed above, including mortality from malignant tumors, infection, respiratory diseases, trauma/accident, or other non-vascular causes. If the cause of death could not be determined based on available information, it was classified as undetermined.

Follow-up was conducted by reviewing and recording information from patient re-admission records, performing telephone interviews with patients or their family members, and verifying death certificates by contacting the respective hospital where the death occurred. All death events were independently adjudicated by two members of the event adjudication committee.

### Statistical methods

In this study, descriptive statistics were first employed to summarize the baseline characteristics of the enrolled elderly patients with CAD and AF. For continuous variables, normality was assessed using appropriate statistical tests. Variables with a normal distribution are reported as mean ± standard deviation, while non-normally distributed variables are presented as median with interquartile range. Categorical variables are described using frequencies and percentages. Comparisons for normally distributed continuous variables were made using the independent samples t-test, and the Mann–Whitney U test was used for non-normally distributed continuous variables. To identify predictors significantly associated with all-cause mortality from a comprehensive set of clinical variables, the Least Absolute Shrinkage and Selection Operator (LASSO) method was employed for variable selection.

All continuous variables were used in their original form without categorization. Prior to LASSO-Cox regression, continuous variables were standardized to a mean of 0 and a standard deviation of 1 to ensure uniform application of the LASSO penalty across variables with different units of measurement. Based on the variables previously selected by LASSO-Cox regression, two predictive models were constructed: (1) Model A: Variables comprising general clinical characteristics + laboratory test indicators. (2) Model B: Variables comprising general clinical characteristics + laboratory test indicators + comorbidity indicators. To assess the robustness of our model and address potential overfitting, we performed 10-fold cross-validation and bootstrap resampling (500 replicates) for internal validation. In the 10-fold cross-validation, we repeated the LASSO-Cox variable selection and model fitting across 10 folds and reported the mean AUC with standard deviation. For bootstrap resampling, we generated 500 bootstrap samples from the training set, refit the model in each sample, and calculated optimism-corrected C-statistics.

Nomograms were developed based on the LASSO-Cox regression models to visualize the prediction of 1-year and 5-year survival probabilities. The predictive performance of the LASSO-Cox models and the CHA_2_DS_2_-VASc score model for all-cause mortality was evaluated and compared using the Area Under the Receiver Operating Characteristic Curve (AUC), calibration curves, and Decision Curve Analysis (DCA). A two-sided *p* value < 0.05 was considered statistically significant.

## Results

### Baseline characteristics of the patients

A total of 2,437 elderly patients (≥65 years) with CAD and AF, hospitalized in the Department of Cardiology at the Chinese PLA General Hospital between January 2010 and December 2017, were initially enrolled in this study. The mean follow-up duration was 5.4 ± 2.5 years, with a follow-up rate of 93.27%. After excluding 4 cases with unspecified time of death, 1,678 patients were included for developing the clinical prediction model for all-cause mortality risk assessment. The mean age of the overall cohort was 75.4 ± 7.1 years (range: 65–96 years). The age distribution was as follows: 65–69 years: 372 (22.2%); 70–74 years: 434 (25.9%); 75–79 years: 448 (26.7%); and ≥80 years: 424 (25.3%). Complete data were available for all variables included in Model B. As summarized in Supplementary Table 1, none of the continuous variables had any missing values in either the training or validation sets. Therefore, all 1,678 patients were included in the final analytical cohort without the need for imputation. These included cases were randomly divided into a training set (*n* = 1,174) and a validation set (*n* = 504) in a 7:3 ratio. Except for unstable angina (37.65% vs. 45.63%, *P* = 0.003), no statistically significant differences were observed in other general clinical data, laboratory findings, or comorbidities between the training and validation sets (*p* > 0.05) (Table 1). In our cohort, ACS accounted for 961 patients (57.3%), while non-ACS/CCS accounted for 717 patients (42.7%). Paroxysmal AF was documented in 776 patients (46.2%), and persistent AF in 266 patients (15.9%). Permanent AF was not recorded in our electronic medical records (Table 2).

**Table 1:** Comparison of general clinical characteristics in elderly patients with CAD and AF.

Clinical characteristics	Training set (*n* = 1174)	Validation set (*n* = 504)	*P* value
Demographics			
Female sex, *n* (%)	499 (42.50)	221 (43.85)	0.648
Age (years), mean ± SD	77.28 ± 6.67	77.56 ± 6.82	0.436
BMI (kg/m2), mean ± SD	24.82 ± 3.79	24.76 ± 3.93	0.814
Laboratory Examinations			
Left ventricular ejection fraction (%), median (Q1, Q3)	57 (52,62)	58 (52.25,63)	0.725
Uric acid (μmol/L), mean ± SD	360.69 ± 121.14	353.91 ± 115.98	0.287
Creatinine (μmol/L), mean ± SD	97.39 ± 58.84	92.83 ± 41.93	0.115
Blood glucose (mmol/L), mean ± SD	6.50 ± 2.56	6.57 ± 2.85	0.653
AST (U/L), mean ± SD	29.82 ± 60.27	28.00 ± 44.23	0.540
ALT (U/L), mean ± SD	26.69 ± 69.33	22.88 ± 29.88	0.235
Plasma fibrinogen (g/L), mean ± SD	3.54 ± 1.05	3.54 ± 0.98	0.923
Plasma D-dimer (μg/mL), mean ± SD	1.24 ± 2.23	1.15 ± 1.85	0.454
Prothrombin time (s), mean ± SD	15.28 ± 6.97	15.09 ± 3.37	0.562
NT-proBNP (pg/mL), median (Q1,Q3)	1202 (47–81.40,2932.25)	1265.50 (569.68,2790.75)	0.621
Platelet count (109/L), mean ± SD	185.48 (62.09)	189.03 (65.20)	0.291
Hemoglobin (g/L), mean ± SD	128.05 ± 19.21	129.38 ± 20.36	0.201
Hematocrit (L/L), mean ± SD	0.38 ± 0.06	0.39 ± 0.06	0.333
Triglycerides (mmol/L), mean ± SD	1.25 ± 0.83	1.19 ± 0.61	0.143
Total cholesterol (mmol/L), mean ± SD	3.83 ± 0.95	3.81 ± 0.92	0.825
LDL (mmol/L), mean ± SD	2.23 ± 0.78	2.26 ± 0.76	0.428
HDL (mmol/L), mean ± SD	1.16 ± 0.35	1.14 ± 0.34	0.256
Type of CAD, *n* (%)			
Unstable angina	442 (37.65)	230 (45.63)	0.003
Stable angina	109 (9.28)	35 (6.94)	0.141
Acute myocardial infarction	86 (7.33)	42 (8.33)	0.540
Ischemic cardiomyopathy	18 (1.53)	7 (1.39)	0.997
Type of Atrial Fibrillation, *n* (%)			
Persistent atrial fibrillation	220 (18.74)	93 (18.45)	0.944
Paroxysmal atrial fibrillation	554 (47.19)	244 (48.41)	0.684
Comorbidities, *n* (%)			
Coronary artery disease	214 (18.23)	90 (17.86)	0.911
Hypertension	886 (75.47)	371 (73.62)	0.457
Dyslipidemia	267 (22.74)	112 (22.22)	0.865
History of myocardial infarction	154 (13.12)	62 (12.30)	0.705
Renal dysfunction	178 (15.16)	67 (13.29)	0.359
Heart failure	452 (38.50)	195 (38.69)	0.985
Diabetes mellitus	354 (30.15)	161 (31.94)	0.502
Stroke	287 (24.45)	118 (23.41)	0.696
Chronic obstructive pulmonary disease	48 (4.09)	17 (3.37)	0.577
Malignant tumor	146 (12.44)	63 (12.50)	1.000
Rheumatic heart disease	44 (3.75)	14 (2.78)	0.395
Valvular heart disease	41 (3.49)	14 (2.78)	0.546

Data are presented as median (interquartile range), mean ± standard deviation, or number (percentage), as appropriate.

AST, aspartate aminotransferase; ALT, alanine aminotransferase; NT-proBNP, N-terminal pro-B-type natriuretic peptide; LDL, low-density lipoprotein; HDL, high-density lipoprotein.

**Table 2:** Distribution of antiplatelet and anticoagulant therapy in the training and validation sets.

Variable	Total (*N* = 1678)	Training set (*N* = 1174)	Validation set (*N* = 504)	*P* value
Antiplatelet agents				
Aspirin	1241 (74.0%)	873 (74.4%)	368 (73.0%)	0.56
Clopidogrel	1052 (62.7%)	735 (62.6%)	317 (62.9%)	0.91
Ticagrelor	38 (2.3%)	26 (2.2%)	12 (2.4%)	0.86
Any antiplatelet	1429 (85.2%)	1000 (85.2%)	429 (85.1%)	0.98
DAPT	892 (53.2%)	619 (52.7%)	273 (54.2%)	0.58
Anticoagulants				
Warfarin	472 (28.1%)	332 (28.3%)	140 (27.8%)	0.84
Rivaroxaban	200 (11.9%)	140 (11.9%)	60 (11.9%)	0.99
Dabigatran	109 (6.5%)	77 (6.6%)	32 (6.3%)	0.89
Any NOAC	309 (18.4%)	217 (18.5%)	92 (18.3%)	0.92
Any anticoagulant	781 (46.5%)	547 (46.6%)	234 (46.4%)	0.95
Combination strategies				
Antiplatelet only	823 (49.0%)	578 (49.2%)	245 (48.6%)	0.83
Anticoagulant only	175 (10.4%)	120 (10.2%)	55 (10.9%)	0.69
Dual therapy	606 (36.1%)	422 (36.0%)	184 (36.5%)	0.84
Triple therapy	254 (15.1%)	171 (14.6%)	83 (16.5%)	0.33

### Selection of relevant factors and model development

**Figure 1:**
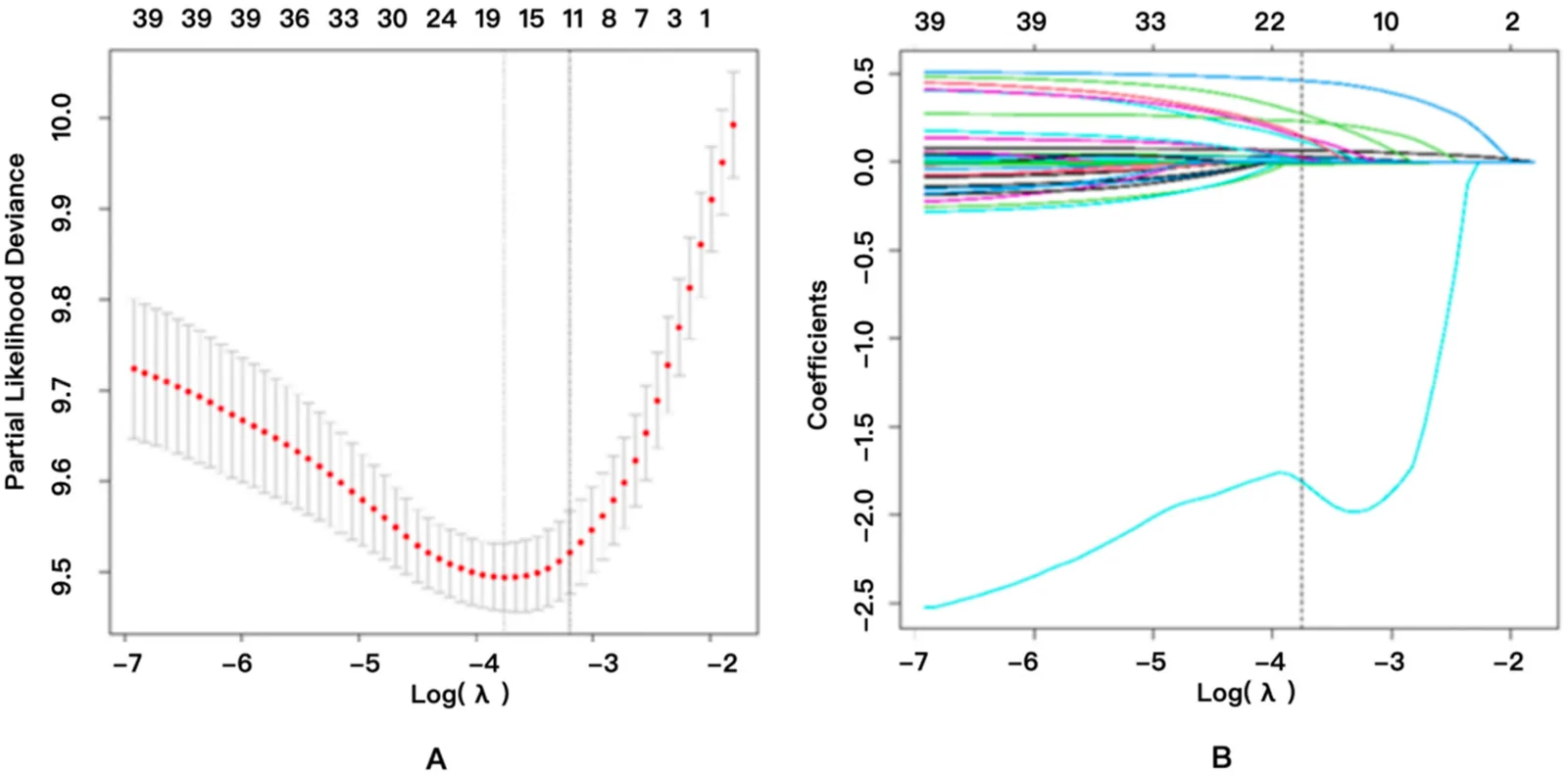
Lasso-Cox regression variable selection via 10-fold cross-validation. **(A)** Relationship between the tuning parameter (log λ) and partial likelihood deviance. The left vertical dashed line indicates log(λ_min) at the minimum partial likelihood deviance, and the right vertical dashed line indicates log(λ_1se) at one standard error from the minimum. The λ_1se criterion was selected to yield a parsimonious model with 17 non-zero coefficients. **(B)** Coefficient profile plot showing the change in variable coefficients against the log(λ) sequence.

Relevant clinical factors associated with all-cause mortality in elderly CAD-AF patients were screened using LASSO-Cox regression. Employing ten-fold cross-validation (Figure 1), the optimal penalty parameter *λ* was selected via 10-fold cross-validation using the λ_1se criterion (the largest λ within one standard error of the minimum deviance), which yielded a parsimonious model with 17 non-zero coefficients. The model ultimately retained 17 variables: age, left ventricular ejection fraction, blood glucose, plasma fibrinogen, plasma D-dimer, prothrombin time, NT-proBNP, hemoglobin, hematocrit, acute myocardial infarction, history of myocardial infarction, renal dysfunction, heart failure, diabetes, stroke, chronic obstructive pulmonary disease, and malignant tumors. The final coefficients of the 17 retained variables are presented in Table 3. Using these variables, two models were constructed:

LASSO-Cox Model A: Included age, left ventricular ejection fraction, blood glucose, plasma fibrinogen, plasma D-dimer, prothrombin time, NT-proBNP, hemoglobin, and hematocrit.
LASSO-Cox Model B: Included all variables from Model A plus acute myocardial infarction, history of myocardial infarction, renal dysfunction, heart failure, diabetes, stroke, chronic obstructive pulmonary disease, and malignant tumors.

**Table 3:** Final coefficients of the 17 variables retained in LASSO-Cox model B.

Variable	Coefficient
Age	0.0797
Left Ventricular Ejection Fraction	−0.0155
Blood Glucose	0.0378
Plasma Fibrinogen	0.0603
D-dimer	0.0543
Prothrombin Time	0.0114
NT-proBNP	1.71 × 10^−5^
Hemoglobin	−0.0031
Hematocrit	−1.6326
Acute Myocardial Infarction	0.4036
History of Myocardial Infarction	0.1011
Renal Insufficiency	0.2984
Heart Failure	0.5273
Diabetes Mellitus	0.2001
Stroke	0.2587
COPD	0.4169
Malignant Tumor	0.4576

**Figure 2:**
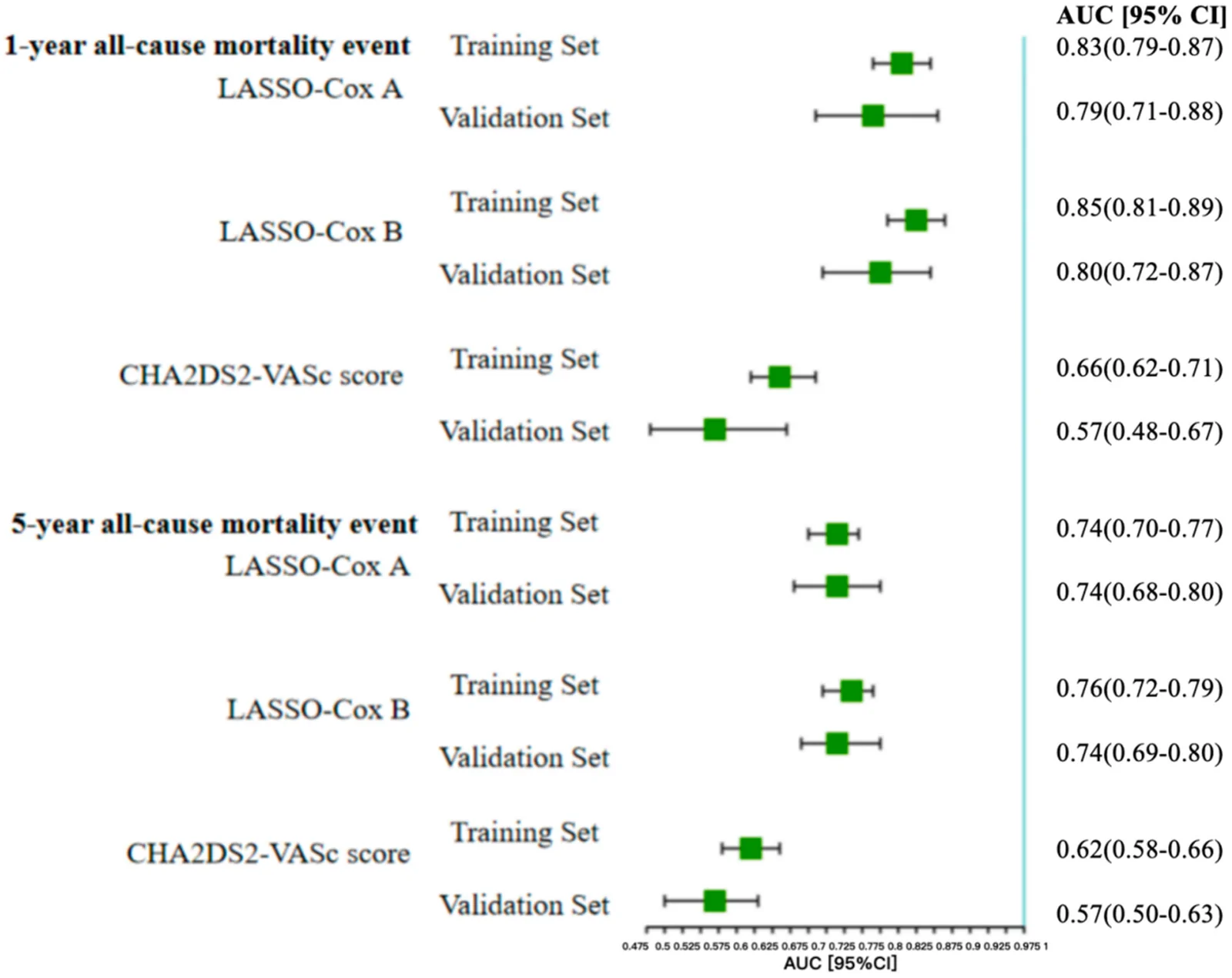
Forest plot of the area under the curve (AUC) for the three models (LASSO-Cox A, LASSO-Cox B, and CHA₂DS₂-VASc score) in the training and validation sets.

The predictive performance of LASSO-Cox Models A and B was described and compared against the CHA_2_DS_2_-VASc score using the Area Under the Curve (AUC) (Figure 2). For predicting 1-year all-cause mortality risk, LASSO-Cox Model B demonstrated the highest predictive performance in the training set (AUC: 0.85, 95% CI: 0.81–0.89). Furthermore, the predictive ability of the CHA_2_DS_2_-VASc score model for 1-year all-cause mortality was significantly lower than that of both LASSO-Cox Model A and Model B, in both the training set (AUC: 0.66, 95% CI: 0.62–0.71) and the validation set (AUC: 0.57, 95% CI: 0.48–0.67). Similarly, for predicting 5-year all-cause mortality risk, the predictive ability of the CHA_2_DS_2_-VASc score model remained significantly inferior to both LASSO-Cox models in both the training set (AUC: 0.62, 95% CI: 0.58–0.66) and the validation set (AUC: 0.57, 95% CI: 0.50–0.63). To further evaluate the robustness of Model B, we performed 10-fold cross-validation. The mean AUC for 1-year mortality prediction was 0.83 (SD: 0.04), and for 5-year mortality was 0.74 (SD: 0.05). Bootstrap resampling (500 replicates) yielded an optimism-corrected C-statistic of 0.679 (95% CI: 0.651–0.708) for Model B. Although this represents a more conservative estimate compared with the validation set AUC (0.78), the 95% confidence interval confirms that the model's discriminative ability remains significantly above chance (0.5). Moreover, the corrected C-statistic still substantially exceeds the CHA_2_DS_2_-VASc score's validation set AUC of 0.57, indicating that the model maintains clinically meaningful predictive performance after accounting for optimism. Since LASSO-Cox Model B exhibited the best predictive performance across both the training and validation sets, it was selected as the final optimal model.

### Construction of a nomogram for predicting all-cause mortality risk in elderly CAD-AF patients

**Figure 3:**
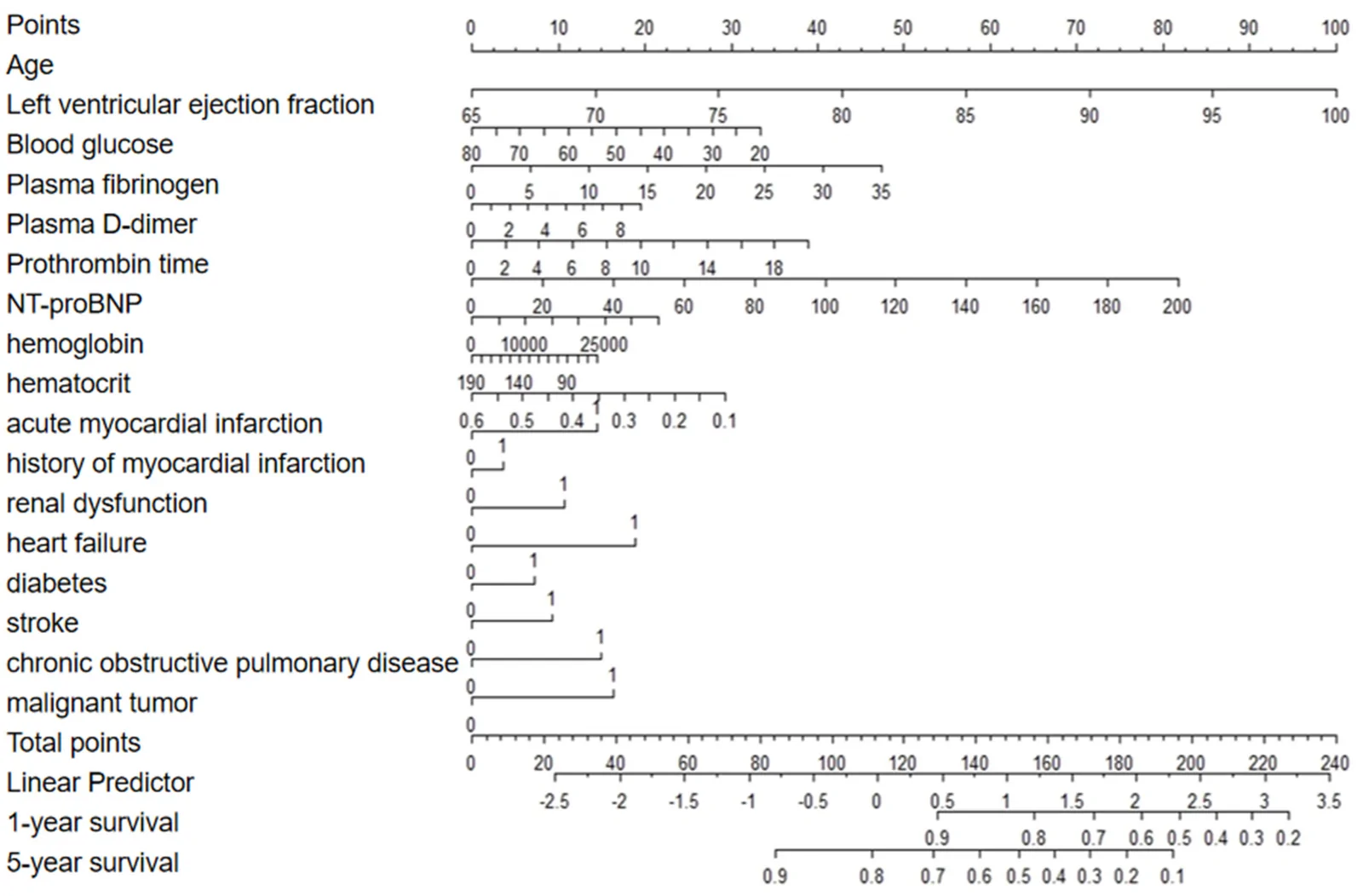
Nomogram for predicting 1- and 5-year all-cause mortality risk in elderly patients with CAD and AF using the Cox proportional hazards regression model.

A Cox proportional hazards regression model was developed based on the 17 independent predictors (LASSO-Cox Model B) in the training set to create a nomogram for predicting the 1-year and 5-year all-cause mortality probabilities in elderly patients with CAD and AF (Figure 3).

Figure 3 shows that age contributed the most points to the nomogram score, followed by prothrombin time, blood glucose, plasma D-dimer, left ventricular ejection fraction, and other variables. For an individual patient, the points corresponding to each predictor can be obtained from the nomogram. The sum of these points yields a total score, which can then be converted into the predicted probability of 1-year and 5-year survival for elderly patients with CAD and AF.

### Evaluation of model performance for predicting all-cause mortality in elderly CAD-AF patients

**Figure 4:**
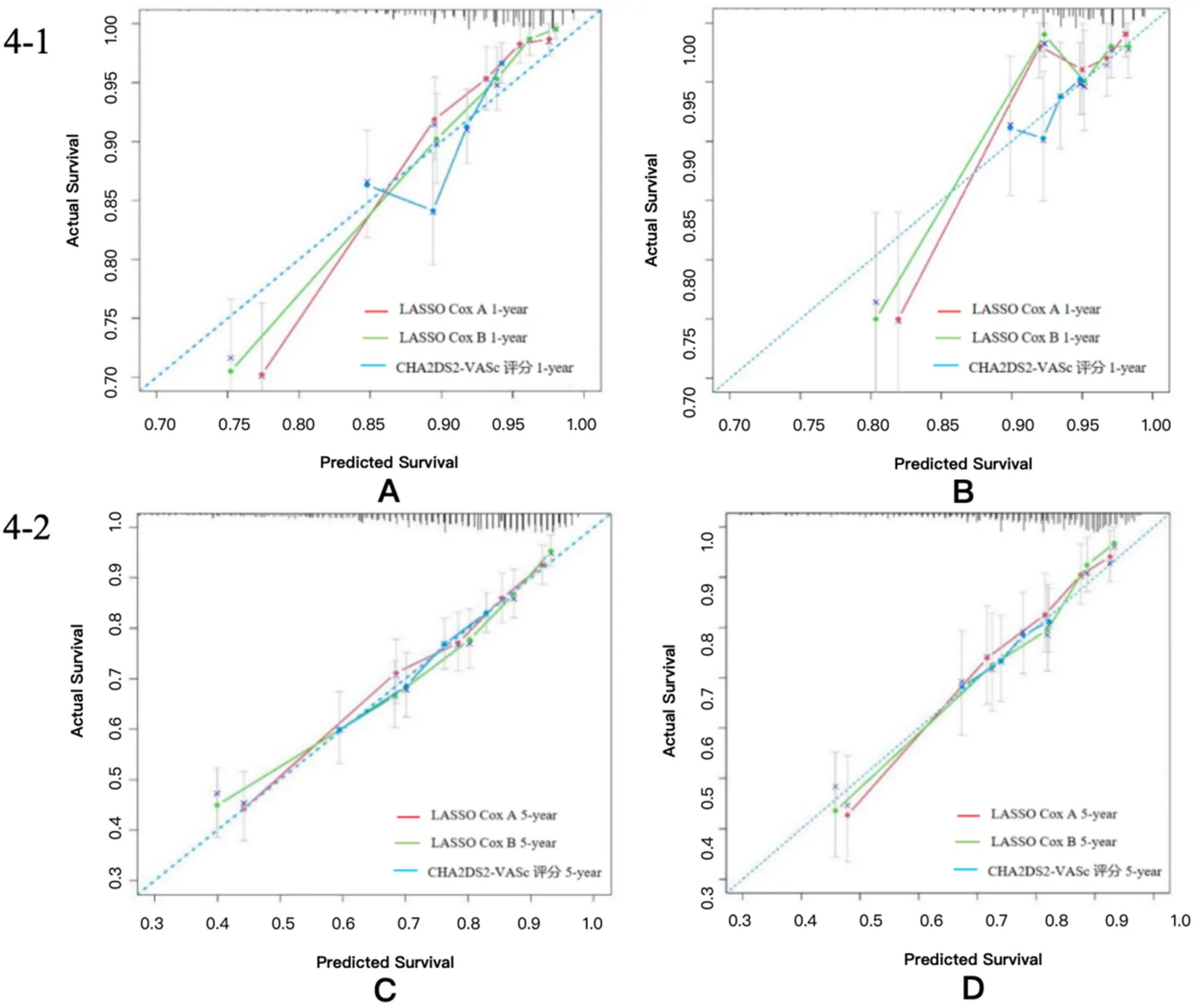
Calibration curves of the Cox proportional hazards regression prediction model for 1-year **(A,B)** and 5-year **(C,D)** all-cause mortality in elderly patients with CAD and AF. Panels **(A,C)** display the calibration curves for the model based on the training set; Panels **(B,D)** show the calibration curves for the model in the validation set. The diagonal dashed line represents the ideal reference line where predictions perfectly match observations. LASSO Cox A: age + left ventricular ejection fraction + blood glucose + plasma fibrinogen + plasma D-dimer + prothrombin time + NT-proBNP + hemoglobin + hematocrit; LASSO Cox B: LASSO Cox A variables + acute myocardial infarction + history of myocardial infarction + renal dysfunction + heart failure + diabetes + stroke + chronic obstructive pulmonary disease + malignant tumor.

The calibration curves of the predictive models are shown in Figure 4. For predicting 1-year all-cause mortality in the training set, all three models demonstrated good agreement with observed outcomes when the predicted survival probability was above 0.9. In the validation set for 1-year all-cause mortality, good agreement was observed across all three models when the predicted survival probability exceeded 0.95. All models also showed favorable calibration for predicting 5-year all-cause mortality in both datasets.

**Figure 5:**
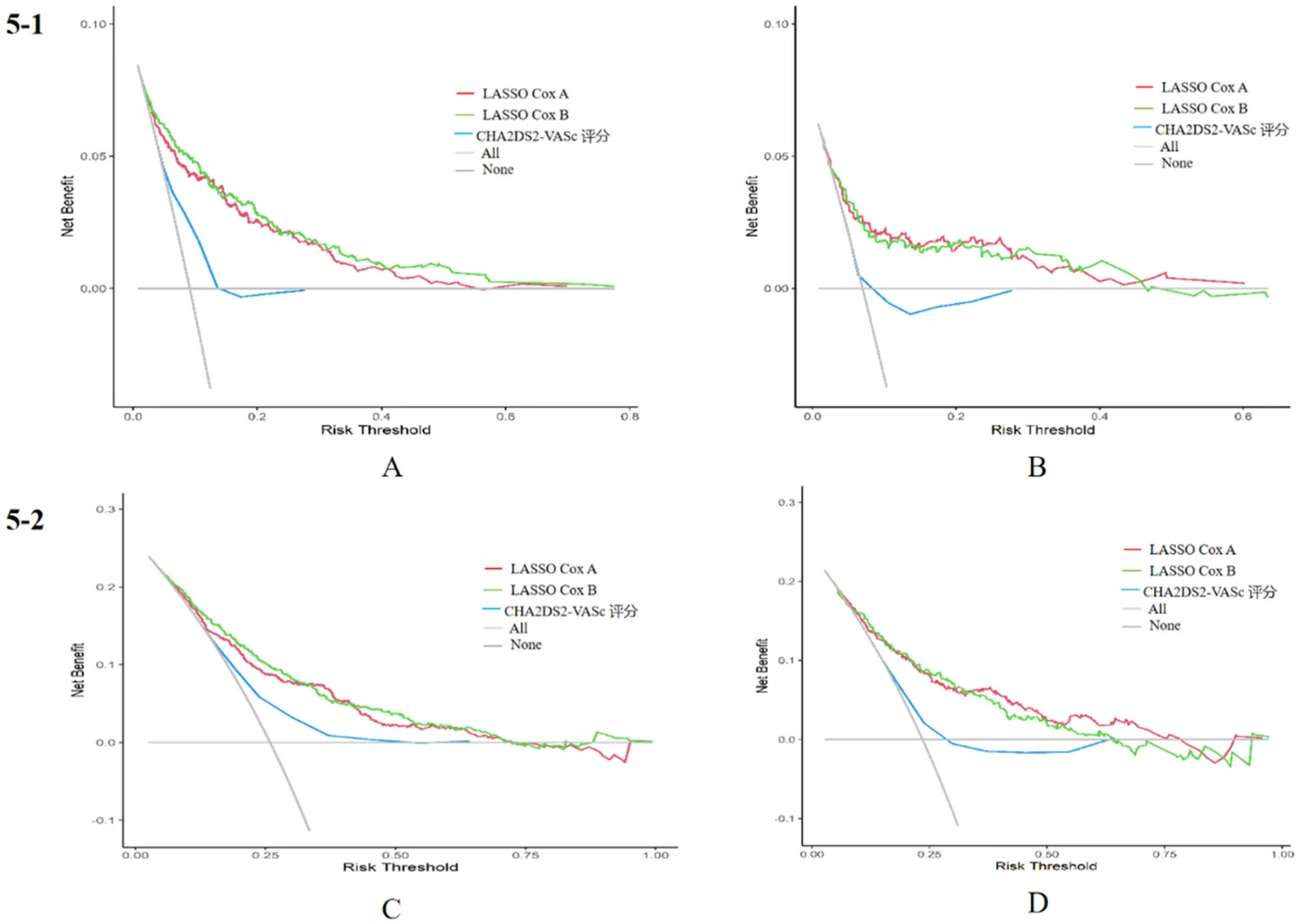
Decision curve analysis of the Cox proportional hazards regression prediction models for 1-year **(A,B)** and 5-year **(C,D)** all-cause mortality in elderly patients with CAD and AF. Panels **(A,C)** show the decision curves for the model based on the training set; Panels **(B,D)** present the decision curves for the model in the validation set. LASSO Cox A: age + left ventricular ejection fraction + blood glucose + plasma fibrinogen + plasma D-dimer + prothrombin time + NT-proBNP + hemoglobin + hematocrit; LASSO Cox B: LASSO Cox A variables + acute myocardial infarction + history of myocardial infarction + renal dysfunction + heart failure + diabetes + stroke + chronic obstructive pulmonary disease + malignant tumor; Model C: CHA_2_DS_2_-VASc score.

DCA revealed that compared to the CHA_2_DS_2_-VASc score model, the LASSO-Cox machine learning models provided a greater net benefit for predicting both 1-year and 5-year all-cause mortality risk (Figure 5).

## Discussion

This study found that a Cox proportional hazards regression model, developed via the LASSO-Cox algorithm and incorporating 17 clinical risk factors, accurately predicted the risk of all-cause mortality in elderly patients with coexisting CAD and AF. Compared to the CHA_2_DS_2_-VASc score model, the prediction model established in this study via the LASSO-Cox machine learning algorithm demonstrated superior discriminatory ability and provided a greater net clinical benefit in both the training and validation sets.

Our cohort demonstrated clinical characteristics broadly comparable to those reported in major elderly Chinese CAD-AF populations. The mean age, prevalence of comorbidities, and distribution of AF subtypes in our study were consistent with findings from the ChiOTEAF registry and other large-scale cohorts, suggesting that our study population is reasonably representative of the general elderly Chinese population with coexisting CAD and AF. There is a lack of machine learning-based prediction models for all-cause mortality risk specifically targeted at elderly patients aged 65 and above with concurrent CAD and AF. This research serves as a valuable reference for machine learning-based risk assessment of all-cause mortality in this specific patient population.

The prediction model developed in this study incorporates three categories of clinical indicators: general patient characteristics, laboratory test results, and patient comorbidities. Our results identified age, left ventricular ejection fraction, blood glucose, plasma fibrinogen, plasma D-dimer, hemoglobin, hematocrit, history of myocardial infarction, history of heart failure, and renal dysfunction as risk factors for all-cause mortality in patients with coexisting CAD and AF. These findings share common ground with the model proposed by Dong Min et al. However, our model additionally includes prothrombin time, NT-proBNP, blood glucose, diabetes, stroke, chronic obstructive pulmonary disease, and malignant tumors as risk factors influencing all-cause mortality in elderly patients with CAD and AF.

Among these, NT-proBNP emerged as the most significant laboratory predictor for all-cause mortality and adverse cardiovascular events. NT-proBNP concentration is associated with left ventricular filling pressure and wall stress (27–29). Its levels increase during left ventricular dysfunction and acute myocardial infarction, and it counteracts heart failure through diuretic, natriuretic, and antihypertensive effects (30, 31). However, the lack of a specific threshold hinders its application in clinical risk prediction (32). Therefore, our prediction model provides a more effective tool for utilizing NT-proBNP in assessing all-cause mortality risk in elderly patients with CAD and AF.

A Swedish study indicated that malignant tumors and renal failure can increase the risk of all-cause mortality in AF patients (33). Elevated blood glucose has also been reported to be associated with adverse cardiovascular events (34, 35). The present study not only corroborates these established associations but further quantifies their predictive value within a dedicated model for elderly patients with CAD and AF. Specifically, variables representing these conditions: malignant tumor, renal dysfunction, and blood glucose were consistently selected into our LASSO-Cox model (Model B) as significant predictors. It underscores that our model successfully captures and integrates these critical laboratory and comorbidity-based risk dimensions. Consequently, greater attention should be paid to laboratory findings and comorbidities in elderly patients with CAD and AF. Early diagnosis and timely intervention targeting these factors are crucial for improving mortality outcomes and prolonging survival.

In a study of 2,174 ACS patients aged 50 and above, Li et al. utilized LASSO regression to select six variables and subsequently developed a Cox regression model to predict 3-year all-cause mortality. Their results showed that this model achieved a predictive performance (AUC) of 0.768 and a calibration of 0.711, both of which were superior to those of the traditional GRACE model (AUC: 0.701; calibration: 0.203) (36). Similarly, Sun et al., working with 468 heart failure patients over 50, applied LASSO regression to select 26 variables and then employed four machine learning algorithms—Extreme Gradient Boosting (XGBoost), Random Survival Forest, Multi-layer Perceptron, and Support Vector Machine—to build models predicting the risk of Major Adverse Cardiovascular Events (MACEs) within six months post-discharge. They found that the model built using the XGBoost algorithm performed best, with an AUC of 0.84 and a calibration of 0.77 (37). These studies collectively affirm the utility of LASSO-based variable selection combined with regression or machine learning modeling in cardiovascular prognosis. Our study extends this methodological approach to a distinct and clinically complex cohort—elderly patients with CAD and AF—a population at notably high risk yet lacking a dedicated prognostic tool. By applying LASSO-Cox regression, we not only confirmed the feasibility of this approach in a multimorbid setting but also developed a model (Model B) that demonstrated robust predictive performance (AUC up to 0.85 for 1-year mortality), thereby providing a tailored risk assessment instrument for this patient group.

The Cox proportional hazards model developed in this study demonstrated significantly superior discriminative ability for all-cause mortality compared to the conventional CHA_2_DS_2_-VASc score in elderly patients with coexisting CAD and AF. We selected the CHA_2_DS_2_-VASc score as the primary comparator for our predictive model. Although originally developed for thromboembolic risk stratification in AF patients, CHA_2_DS_2_-VASc has been extensively validated for predicting all-cause mortality and major adverse cardiovascular events in numerous large-scale cohorts. Its components—age, heart failure, hypertension, diabetes, stroke, and vascular disease—are well-established mortality risk factors. Moreover, as the most widely used risk stratification tool in AF patients, CHA_2_DS_2_-VASc is familiar to all clinicians managing this population. Specifically, our model exhibited robust predictive performance, with an AUC of 0.85 for 1-year mortality in the training set, and maintained stable, clinically relevant accuracy in the independent validation set (1-year AUC: 0.80; sustained AUC>0.75 during long-term follow-up). This performance markedly outperformed the CHA_2_DS_2_-VASc score, which showed limited discriminative power in the same cohort. More importantly, decision curve analysis confirmed that our model, which integrates over ten clinical risk factors selected via the LASSO-Cox algorithm, provides a greater net clinical benefit across a wide range of risk thresholds. These findings collectively indicate that a multivariate model incorporating diverse clinical, laboratory, and comorbidity data offers a more accurate and clinically useful risk stratification tool for this high-risk, complex population than the traditional score-based approach. We selected the CHA_2_DS_2_-VASc score as the primary comparator for our predictive model. Although originally developed for thromboembolic risk stratification in AF patients, CHA_2_DS_2_-VASc has been extensively validated for predicting all-cause mortality and major adverse cardiovascular events in numerous large-scale cohorts. Its components—age, heart failure, hypertension, diabetes, stroke, and vascular disease—are well-established mortality risk factors. Moreover, as the most widely used risk stratification tool in AF patients, CHA_2_DS_2_-VASc is familiar to all clinicians managing this population.

The choice of all-cause mortality as the primary endpoint warrants justification. In elderly patients with CAD and AF, who carry a high burden of competing comorbidities including malignancy, renal insufficiency, and COPD, clinicians and patients are primarily concerned with overall survival rather than cause-specific outcomes. Unlike cause-specific mortality, which is subject to misclassification and adjudication variability—particularly in retrospective studies where death certificates may be incomplete—all-cause mortality is an objective, hard, and unbiased endpoint. Moreover, the CHA_2_DS_2_-VASc score, the most widely used risk stratification tool in AF patients, has been extensively validated for all-cause mortality prediction, supporting the clinical relevance of this endpoint. We acknowledge that predictors may differ between cardiovascular and non-cardiovascular death; however, in this multimorbid population, overall survival remains the ultimate clinical metric of interest. Regarding the competing risk issue, we acknowledge that non-cardiovascular death competes with cardiovascular death. While we could not perform a formal Fine-Gray competing-risk analysis due to the lack of reliable cause-specific data, we note that the predictors identified in our model—age, NT-proBNP, heart failure, renal insufficiency, malignant tumor, and COPD—are well-established risk factors for both cardiovascular and non-cardiovascular mortality. This suggests that our model captures a broad spectrum of mortality risk that is clinically meaningful regardless of the specific cause. The heterogeneity of CAD and AF subtypes deserves careful consideration. While our cohort included patients with various clinical presentations, we were unable to perform formal subgroup analyses due to inconsistent documentation of subtype classification in our retrospective electronic medical records, particularly during the earlier years of the study period. Additionally, certain subgroups—such as persistent AF and specific CAD phenotypes—were represented by relatively small numbers, precluding robust statistical estimation. We therefore tested for interactions between key clinical indicators and model predictors; none reached statistical significance, providing some reassurance that the predictive effects are broadly consistent across major clinical presentations. However, we acknowledge this as an important limitation and emphasize that future prospective studies with standardized subtype documentation are needed to validate our model across different CAD and AF phenotypes.

### Limitations

This study has several limitations. First, the types of variables available in the clinical follow-up database for model selection were limited. A lack of structured information, such as details regarding surgical interventions and imaging data, restricted the inclusion of other predictors that might significantly influence clinical outcomes.

Secondly, the study exclusively employed the LASSO-Cox machine learning algorithm for model construction. The randomness in variable selection can differ across various machine learning algorithms. Therefore, future work should involve comparing models built using other algorithms, including Random Forest, to further validate and refine our approach. We did not perform formal testing of the proportional hazards assumption using Schoenfeld residuals, as our model was developed using penalized Cox regression, which does not natively support this diagnostic. While our primary focus was on prognostic prediction rather than causal inference, and calibration curves and DCA demonstrated good model performance, we acknowledge this as a limitation. The single-center design of this study limits the generalizability of our findings. Although our cohort shared clinical characteristics with other major elderly CAD-AF populations, patients treated at a single tertiary referral center may differ from those in community hospitals or primary care settings. While we used rigorous internal validation (7:3 split, 10-fold cross-validation, and bootstrap resampling) and a relatively large sample size to enhance robustness, external validation in independent, multi-center cohorts is essential before our model can be confidently implemented in routine clinical practice. Due to the retrospective nature of our data and the high proportion of deaths occurring outside of hospital settings, reliable cause-specific mortality data were not available for a substantial number of patients. We therefore used all-cause mortality as the primary endpoint. While this endpoint is objective and clinically relevant, it does not allow differentiation of risk factors by specific death causes, and the absence of competing-risk analysis represents a limitation. The lack of external validation represents a major limitation of our study. While we performed 10-fold cross-validation and bootstrap resampling to assess internal validity, external validation in an independent cohort is essential to confirm the generalizability of our findings. The single-center design and the 2010-2017 study period further limit the applicability of our model to contemporary, multi-center settings. We acknowledge that predictors for cardiovascular and non-cardiovascular death may differ. However, in elderly patients with CAD and AF, overall survival is the ultimate concern for clinicians and patients, and all-cause mortality is widely accepted as a robust and unbiased endpoint in prognostic studies. Future prospective studies with standardized cause-of-death adjudication are needed to validate our findings for cardiovascular and non-cardiovascular mortality separately.

Several considerations regarding antithrombotic therapy merit discussion. We extracted detailed antiplatelet and anticoagulation data and included them as candidate predictors in our LASSO-Cox variable selection. However, none of the antithrombotic treatment variables were retained in the final model. We attribute this primarily to collinearity with indication-defining comorbidities. In clinical practice, antithrombotic prescriptions are guideline-driven and strongly correlated with underlying conditions such as prior myocardial infarction, heart failure, and stroke. These comorbidities were already selected into Model B, and LASSO regression preferentially retained the disease indicators over the treatments. We acknowledge that several geriatric-specific variables were not available in our dataset. Frailty, functional status, cognitive impairment, nutritional status, falls risk, polypharmacy, and comprehensive multimorbidity burden are well-recognized prognostic factors in elderly populations. However, these measures were not routinely documented in our retrospective cardiology cohort. While we were unable to include these variables, our model does capture certain correlates of frailty—including age, comorbidities (heart failure, renal insufficiency, COPD, malignant tumor), and laboratory parameters (hemoglobin, NT-proBNP)—which may partially reflect the patient's overall health status. Nevertheless, the absence of dedicated geriatric assessment tools represents an important limitation. Future prospective studies incorporating comprehensive geriatric evaluation are needed to determine whether these additional measures enhance the predictive performance of our model.

We acknowledge that several geriatric-specific variables were not available in our dataset. Frailty, functional status, cognitive impairment, nutritional status, falls risk, polypharmacy, and comprehensive multimorbidity burden are well-recognized prognostic factors in elderly populations. However, these measures were not routinely documented in our retrospective cardiology cohort. While we were unable to include these variables, our model does capture certain correlates of frailty—including age, comorbidities (heart failure, renal insufficiency, COPD, malignant tumor), and laboratory parameters (hemoglobin, NT-proBNP)—which may partially reflect the patient's overall health status. Nevertheless, the absence of dedicated geriatric assessment tools represents an important limitation. Future prospective studies incorporating comprehensive geriatric evaluation are needed to determine whether these additional measures enhance the predictive performance of our model.

## Conclusion

The Cox proportional hazards regression model established based on the machine learning algorithm demonstrated superior predictive ability for all-cause mortality risk in elderly patients with CAD and AF, showing both higher predictive performance and greater net benefit compared to the CHA_2_DS_2_-VASc score model.

## Data Availability

The data analyzed in this study is subject to the following licenses/restrictions: The dataset contains potentially sensitive patient information from electronic medical records and is subject to institutional data protection policies. It is not publicly available due to privacy and ethical restrictions. Access may be granted to qualified researchers upon reasonable request to the corresponding author, subject to approval from the institutional ethics committee. Requests to access these datasets should be directed to yintong301@163.com.
